# Discovery of Tick-Borne Karshi Virus Implies Misinterpretation of the Tick-Borne Encephalitis Virus Seroprevalence in Northwest China

**DOI:** 10.3389/fmicb.2022.872067

**Published:** 2022-05-03

**Authors:** Yuan Bai, Yanfang Zhang, Zhengyuan Su, Shuang Tang, Jun Wang, Qiaoli Wu, Juan Yang, Abulimiti Moming, Yujiang Zhang, Lesley Bell-Sakyi, Surong Sun, Shu Shen, Fei Deng

**Affiliations:** ^1^State Key Laboratory of Virology and National Virus Resource Centre, Wuhan Institute of Virology, Chinese Academy of Sciences, Wuhan, China; ^2^University of Chinese Academy of Sciences, Beijing, China; ^3^Center for Disease Control and Prevention of Xinjiang Uygur Autonomous Region, Urumqi, China; ^4^Department of Infection Biology and Microbiomes, Institute of Infection, Veterinary and Ecological Sciences, University of Liverpool, Liverpool, United Kingdom; ^5^Xinjiang Key Laboratory of Biological Resources and Genetic Engineering, College of Life Science and Technology, Xinjiang University, Urumqi, China

**Keywords:** Karshi virus, tick-borne encephalitis virus, prevalence, serological correlation, cross-reaction

## Abstract

Despite few human cases of tick-borne encephalitis virus (TBEV), high rates of TBEV seroprevalence were reported among humans and animals in Xinjiang Uygur Autonomous Region in Northwestern China. In this study, the Karshi virus (KSIV) was identified and isolated from *Hyalomma asiaticum* ticks in Xinjiang. It belongs to the genus *Flavivirus* of the family *Flaviviridae* and is closely related to TBEV. KSIV infects cell lines from humans, other mammals and ticks, and causes encephalitis in suckling mice. High minimum infection rates (4.96%) with KSIV were detected among tick groups. KSIV infections have occurred in sheep and marmots, resulting in antibody-positive rates of 2.43 and 2.56%, respectively. We further found that, of the KSIV antibody-positive serum samples from animals, 13.9% had TBEV exposure showing cross-reaction to KSIV, and 11.1% had KSIV infection resulting in cross-reaction to TBEV; 8.3% were likely to have co-exposure to both viruses (or may be infected with one of them and present cross-reactivity with the other). The results revealed a substantial KSIV prevalence among ticks in Xinjiang, indicating exposure of animals to KSIV and TBEV. The findings implied misinterpretation of the high rates of TBEV seroprevalence among humans and animals in previous studies. There is a need to develop detection methods to distinguish KSIV from TBEV and to perform an in-depth investigation of KSIV and TBEV prevalence and incidence in Northwestern China, which would enhance our preparation to provide medical treatment of emerging diseases caused by tick-borne viral pathogens such as KSIV.

## Introduction

Ticks are blood-sucking arthropods and vectors of viral pathogens causing diseases in animal hosts and humans. Located in the Northwest of China, Xinjiang Uygur Autonomous Region is the habitat of 49 tick species (Zhang et al., 2019), and is known as a natural focal point of tick-borne viral diseases, such as Crimean–Congo haemorrhagic fever and tick-borne encephalitis (CCHF and TBE, also known in China as Xinjiang Haemorrhagic Fever and Forest Encephalitis, respectively). The first record of a CCHF outbreak occurred in Bachu County of Xinjiang in 1965, resulting in a fatality rate of up to 50% (Papa et al., 2002). Since then, outbreaks and sporadic cases among the residents were reported from different areas of Xinjiang. Although no cases have been further reported after 2003, the causative agent, Crimean–Congo haemorrhagic fever virus, could be persistently detected and isolated from ticks collected in different locations there (Sun et al., 2009; Guo et al., 2017; Moming et al., 2018; Zhang et al., 2018). While there were very few cases of TBE disease diagnosed in Xinjiang, serological evidence revealed that the residents had tick-borne encephalitis virus (TBEV) exposure (Zhang et al., 2013). High positive rates of TBEV were detected from the ticks *Ixodes persulcatus* (minimum infection rate: 14.3–47.7%) and *Dermacentor silvarum* (minimum infection rate: 0.01–1.67%) in Xinjiang, where both Siberian and Far Eastern TBEV strains were isolated from these two tick species (Xie et al., 1991; Wang H. et al., 2015; Liu et al., 2016; Zhang G. et al., 2017). These studies showed substantial prevalence of Crimean–Congo haemorrhagic fever virus and TBEV, which posed persistent risks from virus transmission and infection to humans and animals in Northwestern China.

Novel tick-borne viruses (TBVs) have been discovered and isolated from ticks in Xinjiang in the recent decade. Guertu virus (GTV, belonging to the genus *Bandavirus* in the family *Phenuiviridae*) was isolated from *Dermacentor nuttalli* and was considered a potential pathogen, as evidenced by local people having neutralizing antibodies to this virus (Shen et al., 2018). Tamdy virus (TAMV, belonging to the genus *Orthonairovirus* in the family *Nairoviridae*) was detected and isolated from *Hyalomma asiaticum* in Xinjiang and might be related to a local disease outbreak among humans as early as 1997 (Moming et al., 2021). A variety of novel viruses was found in ticks by next-generation sequencing (Li et al., 2015); however, risks of their transmission from ticks to humans and other animals were unclear. Recently, one of these viruses identified by sequencing, Tacheng tick virus (TcTV-1), was identified as the causative agent of fever in a patient in Xinjiang (Liu et al., 2020). This raised concern about the medical significance of novel TBVs and suggested the urgent need to identify novel tick-borne viral pathogens and to perform further in-depth surveys on the prevalence and potential threats of TBVs in Xinjiang, China.

Tick-borne encephalitis virus is the representative virus of the “TBEV serocomplex” group in the genus *Flavivirus*, family *Flaviviridae*. This group is composed of the TBEV-related viruses transmitted by ticks, including the human disease-related pathogens TBEV, Omsk haemorrhagic fever virus, Powassan virus and Kyasanur Forest disease virus, and zoonotic pathogens such as louping ill virus, Langat virus, and Royal Farm virus (Grard et al., 2007). Karshi virus (KSIV), which was firstly isolated from soft ticks from Uzbekistan in 1972 (Lvov et al., 1976), also belongs to this group and is genetically related to TBEV. However, its prevalence among ticks and pathogenicity for domestic animals and humans remained unclear.

In this study, KSIV was identified in and isolated from *Hy. asiaticum* ticks in Xinjiang, China. We characterized the etiological characteristics of KSIV and the pathogenesis in mice and evaluated its potential to infect humans and other animals by surveying KSIV prevalence among ticks and serological exposure in animals. Moreover, serological cross-reaction between KSIV and TBEV was demonstrated using laboratory-prepared polyclonal antibodies and serum samples from domestic and wild mammals. The results revealed a substantial prevalence of KSIV in ticks and the occurrence of KSIV infection among animals, which suggested the threat of KSIV spillover from ticks and animal hosts to humans. The serological cross-reactivity between these two viruses raised the need to develop detection methods to distinguish KSIV from TBEV, which would facilitate follow-up investigations of these two viruses and promote subsequent evaluation of the threat from KSIV infection to humans in Northwestern China.

## Materials and Methods

### Viruses and Cell Lines

The detailed information regarding human, other mammal, tick and mosquito cell lines used in this study, and the TBEV strain NM-Tick-2020 used to investigate its serological correlation with KSIV, are described in Supplementary Material.

### Tick Collection and Viral Metagenomics Analysis

Ticks were collected from vegetation at fourteen sites in cities and counties of Xinjiang during 2016–2017. Tick species were identified by PCR amplification of a partial sequence of the ribosome Internal Transcribed Spacer 2 (ITS2) and confirmed by Sanger sequencing as previously described (Ya et al., 2016). These ticks were grouped (*n* = 50–100/group) according to species and sampling locations. Clarified homogenates of each tick group were prepared and inoculated into Kunming suckling mice (1–2 days old) by both intraperitoneal and intracranial routes as previously described (Deng et al., 2007). Diseased mice were sacrificed, and their brains were collected and preserved in glycerine at −80°C until further examination. Homogenates of the brain tissue from one diseased mouse randomly selected from a group were prepared and sub-packaged as described (Zhang Y. et al., 2017). Total RNA was purified from the brain homogenates (300 μL) and subsequently applied for next-generation sequencing (RNAseq) using the Illumina Hiseq 3000 platform according to the manufacturer’s instructions (Illumina, San Diego, CA, United States). The sequencing data was subjected to quality control (FastaQC, Trimmotic), then the Trinity (Version 2.5) program was used for sequence assembly and splicing. BLASTn and BLASTx comparisons were performed to discover and annotate virus-related sequences.

### Virus Isolation, Infection Assays, and One-Step Growth Curve Analyses

The KSIV-positive brain homogenates (200 μL) from a diseased mouse were diluted and incubated with BHK-21 cells. Further subculture was performed for four generations as previously described (Zhang Y. et al., 2017). To obtain the 5′ and 3′ ends of the KSIV genomic sequence, rapid amplification of cDNA ends (RACE) was performed with specific primers using a SMARTer RACE cDNA Amplification Kit (Takara, Kusatsu, Japan). Virus infection in cells was examined by immunofluorescence assay (IFA; Supplementary Material), and plaque assay was performed as previously described (De Madrid and Porterfield, 1969). The KSIV particles were purified from cell culture supernatants and were visualized using a transmission electron microscope (TEM, Tecnai G2 20 TWIN, United States) as previously described (Zhang Y. et al., 2017). Sections of BHK-21 cells infected with KSIV were prepared and examined by TEM to visualize the intracellular distribution of KSIV (Moming et al., 2021).

The KSIV infection assays on human, other mammal, tick and mosquito cell lines were performed at a dose of 1 multiplicity of infection (MOI) unit per cell as previously described (Shen et al., 2018). IFAs were performed to examine KSIV infection in cells at 48 h post-infection (h p.i). KSIV growth properties were characterized by one-step growth curve analysis in SH-SY5Y, SW-13, and BHK-21 cells as previously described (Dai et al., 2018), and titers were determined by end-point dilution assays on BHK-21 cells.

### Animal Experiments

To investigate KSIV pathogenesis, adult mice and suckling mice were inoculated with KSIV and monitored for signs of disease onset and changes in body weight as well as survival rates. Tissues were harvested from suckling mice after KSIV infection. A qRT-PCR was performed to quantify KSIV RNA copies in tissues. H&E staining, immunohistochemical assays and IFAs were performed on tissue sections to characterize KSIV infection in the brains of suckling mice. The processes of each assay are detailed in Supplementary Material.

### Serological Tests for Karshi Virus Among Domestic and Wild Animals

Serum samples collected from sheep and small mammals in Xinjiang during 2006–2015 were archived in the National Virus Resource Center, Wuhan. IFAs were performed to determine the antibody response to KSIV among the samples. The IFA-positive sera were further verified by western blot using purified KSIV particles. The levels of antibody response to E protein domain III (E-DIII) of KSIV were measured by Luciferase Immunoprecipitation System (LIPS) assays. Neutralizing activity of the antibody-positive serum samples against KSIV was determined by virus neutralization test (VNT). All the above methods are detailed in the Supplementary Material.

### Analyses of Serological Cross-Reactivity Between Karshi Virus and Tick-Borne Encephalitis Virus

Immunofluorescence assay (IFAs) were carried out to determine the efficiencies of polyclonal antibodies, prepared in-house against KSIV E protein (α-KSIV-E) and TBEV E protein (α-TBEV-E), reacting to BHK-21 cells infected with each of the two viruses. Images were captured and reaction efficiency values were expressed as the percentage of Harmony High-Content Imaging and Analysis Software-defined (PerkinElmer, Inc., United States) fluorescence-labeled cells among the total number of cells in each test for each of the dilutions. The 50 and 90% detection efficiencies (DE_50_ and DE_90_) of α-KSIV-E and α-TBEV-E in reaction to KISV and TBEV antigens were calculated, respectively, using the least square fitting method in GraphPad Prism (Version 8).

Cross-neutralization between KSIV and TBEV was characterized by performing virus neutralization tests using KSIV- or TBEV- inoculated mouse serum. The neutralizing serum dilution was determined as an antibody titer that inhibited 50 and 90% of viral infection (VNT_50_ and VNT_90_).

The serological reaction of field-collected KSIV antibody-positive serum samples to TBEV antigens were further investigated by IFAs, western blot, and LIPS as well as neutralization assays against TBEV. All the above-mentioned materials and methods are detailed in the Supplementary Material.

### Sequencing, Bioinformatics, and Data Analysis

The complete genome sequence of KSIV strain WJQ-16209 [GenBank No: MH688511; China National GeneBank DataBase (CNGBdb) No: CNP0002450], and partial sequences representing other KSIV strains (GenBank Nos: OL699896–OL699904, MH688625, and MH688632) detected in ticks by RT-PCR were deposited in CNGBdb or GenBank. The data resulting from RNAseq of the brain homogenates from diseased mice were deposited in the CNGBdb (No: CNP0002610).^1^

All sequence alignments were performed using MEGA X. The phylogenetic tree of flaviviruses was built based on the full-length nucleic acid sequence of the open reading frame (ORF) and the partial sequences (302 nucleotides, nt) of the NS5 protein sequence using the maximum likelihood method with a bootstrap value of 1,000. The alignment of E proteins of KSIV (KSIV-E) and TBEV (TBEV-E) was illustrated using ESPript 3.0^2^ to predict the secondary structure of KSIV-E according to that of TBEV-E. The predicted three-dimensional (3D) structure of KSIV-E protein was constructed using I-TASSER (Zheng et al., 2021) and visualized using Pymol™ (version 1.4.1). The significant difference of data between groups was calculated using Student’s *t*-test.

### Biosafety and Ethics

The *in vitro* and *in vivo* experiments involving KSIV were conducted in a Biosafety Level 2 (BSL-2) laboratory and an Animal Biosafety Level 2 (ABSL-2) laboratory, respectively, while the *in vitro* and *in vivo* experiments involving TBEV were conducted in BSL-3 and ABSL-3 laboratories, according to the Directory of Pathogenic Microorganisms Transmitted among Humans issued by the Chinese Ministry of Health.^3^ Animal experiments were approved by the ethics committee of Wuhan Institute of Virology, Chinese Academy of Sciences under the approval numbers WIVA33201901 and WIVA33202108.

## Results

### Karshi Virus Was Identified in and Isolated From *Hyalomma* Ticks in Xinjiang, China

**FIGURE 1:**
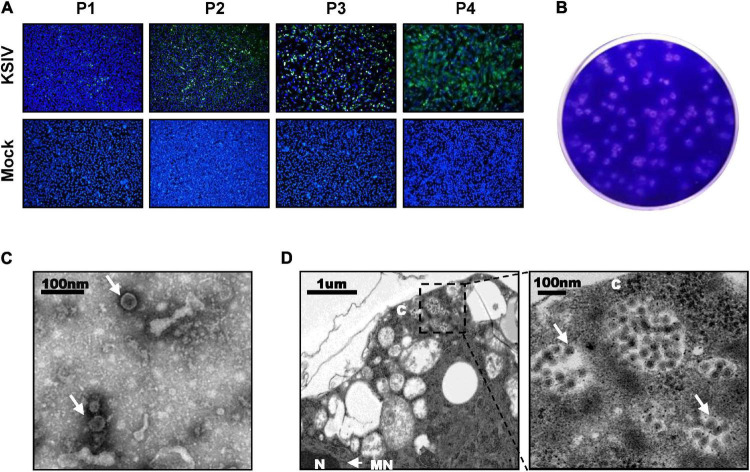
Karshi virus (KSIV) isolation, characteristics and analysis of viral particles. **(A)** Immunofluorescence assays to survey KSIV isolation in BHK-21 cells. The images were taken from different passages showing KSIV proliferation. Cells were immunostained and green fluorescence indicates the cells infected by KSIV. P1, the first passage; P2, the second passage; P3, the third passage; P4, the fourth passage. **(B)** Plaque assay to verify KSIV isolation in BHK-21 cells with the P4 supernatants. Cells were stained with crystal violet to show plaques at 3 days post-infection. **(C)** TEM analysis of KSIV particles purified from supernatants harvested from BHK-21 cells infected with KSIV at 3 days post-infection. The clarified supernatants were subjected to ultracentrifugation. The fractions containing viral particles were harvested and used in negative-staining. **(D)** The image obtained by TEM shows that virus particles were located in the cytoplasm of infected BHK-21 cells. The enlarged area on the right shows groups of virus particles (arrow). N, nucleus; C, cytoplasm; NM, nuclear membrane.

A total of 31,542 ticks were collected, including the species *Hyalomma detritum*, now reclassified as *Hyalomma scupense* (Apanaskevich et al., 2010; *n* = 326), *Hy. asiaticum* (*n* = 8,741) and *D. nuttalli* (*n* = 22,475). To isolate viruses from ticks, the clarified homogenates prepared from tick groups were inoculated into suckling mice, which were monitored for disease onset within 14 days after inoculation. One group of mice, which were inoculated with the homogenates from a group of *Hy. asiaticum* ticks (*n* = 400) collected from Wujiaqu City in 2016, developed clinical illness. Brains were harvested from diseased mice and homogenized for the second round of inoculation, during which illness among suckling mice was still noted, suggesting potential pathogen(s) might be isolated and could be effectively passaged among generations of suckling mice. Subsequently, a total number of non-redundant 92,253,190 reads were obtained by RNAseq from one diseased mouse brain in the second round of inoculation. The sequence comparison analysis showed that 37,872 reads were closely related to the flavivirus KSIV, which had never before been found in China. KSIV RNA present in the brain of the diseased mice was confirmed by RT-PCR on the rest of the homogenates (data not shown). The homogenate of this KSIV-positive brain sample was diluted and further incubated with BHK-21 cells. As suggested by the results of IFAs, KSIV was isolated and amplified after passages on BHK-21 cells, as increasing infection levels were observed in cells from the first passage (P1) to the fourth (P4) (Figure 1A). KSIV infection could also induce plaque formation in BHK-21 cells (Figure 1B). The TEM analysis showed that KSIV is an enveloped particle with a diameter of approximately 50 nm (Figure 1C). KSIV particles were mainly located in cytoplasmic vesicles of BHK-21 cells (Figure 1D). The full-length genomic sequence of a new KSIV strain (10,828 nt), which contains a polyprotein ORF (10,251 nt), was obtained by sequence assembly based on the KSIV-related reads together with the result from RACE. This new strain clusters with three other KSIV strains reported from Central Asian countries (Supplementary Figure 1), and shares 85.7–86.9% nucleotide sequence identity and 94.5–95.1% amino acid similarity to them (Supplementary Table 1). It was therefore designated as strain WJQ16209 by following the year and location of tick sampling. As a member of the TBEV serocomplex group belonging to the genus *Flavivirus* in the family *Flaviviridae*, the polyprotein ORF of KSIV strain WJQ16209 has 69.7–71.7% amino acid and 67.1–67.9% nucleotide identity to those of other viruses in this group, with the highest to that of TBEV (67.9% for nucleotide and 71.1% for amino acid sequences) (Supplementary Table 1).

### Human, Other Mammal, Tick, and Mosquito Cell Lines Had Different Levels of Susceptibility to Karshi Virus

**FIGURE 2:**
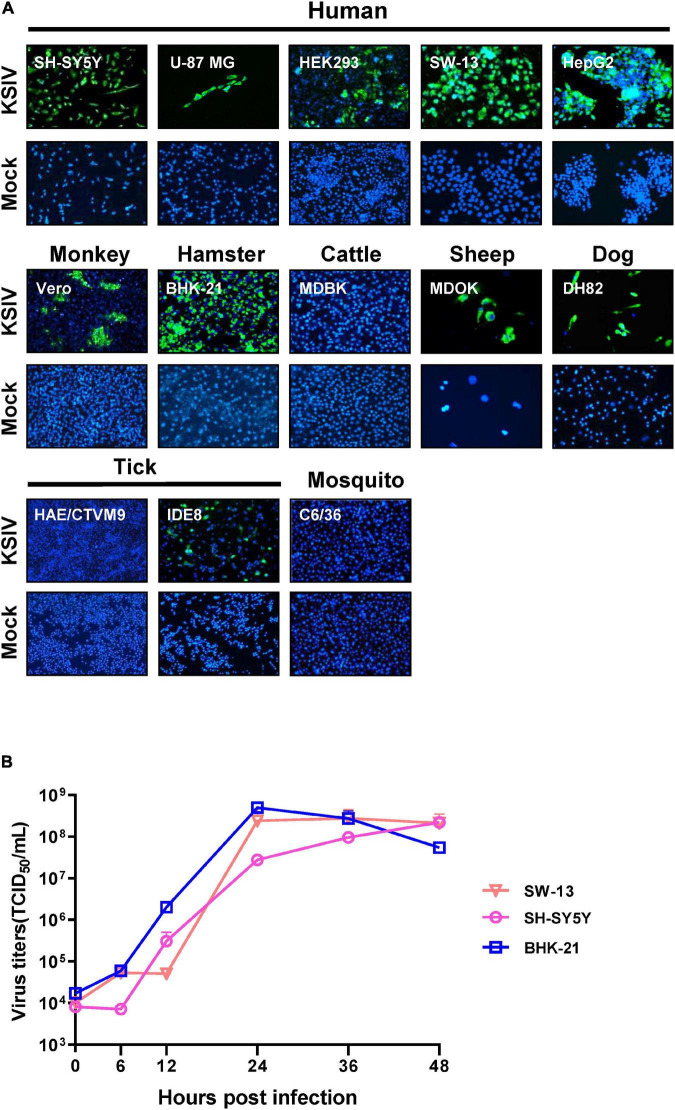
The susceptibility of cell lines derived from different hosts to KSIV and viral one-step growth curve test. **(A)** Cell lines derived from humans (SH-SY5Y, U-87 MG, SW-13, HEK239 and HepG2), other mammals (Vero, BHK-21, MDBK, MDOK, and DH82), mosquito (C6/36) and ticks (HAE/CTVM9 and IDE8) were infected with KSIV at MOI of 1. The cells were cultured for 48 h and examined by immunofluorescence for KSIV E protein. KSIV infection was detected in all cell lines except MDBK, HAE/CTVM9 and C6/36. **(B)** The viral one-step growth curve in various cell lines. BHK-21 cells were infected with KSIV at an MOI of 5 and the supernatant for collected infection of cell lines of different species: human (SH-SY5Y, SW13) and hamster (BHK-21), virus titers were measured by end-point dilution assays.

As evidenced by the green fluorescent foci present in infected cells in IFAs at 48 h p.i (Figure 2A), KSIV can infect human cell lines, resulting in high efficiencies in neuroblastoma cells (SH-SY5Y), adrenocortical carcinoma cells (SW-13) and liver hepatocellular carcinoma cells (HepG2) and lower efficiency in HEK293 (embryonic kidney cells) and U-87 MG (malignant glioblastoma cells) (Figure 2A, upper panel). KSIV can infect other mammalian cell lines including Vero (monkey), MDOK (sheep), and DH82 (dog) (Figure 2A, middle panel). However, the infection efficiencies in these cells were lower than in BHK-21 (hamster), which had green fluorescence in almost all cells, while KSIV infection was not detected in the bovine cell line (MDBK) (Figure 2A, middle panel). Since KSIV was isolated from *Hy. asiaticum* ticks, its infection in tick cells was characterized using the HAE/CTVM9 (*Hyalomma anatolicum*) and IDE8 (*Ixodes scapularis*) cell lines. At 48 h p.i., green fluorescence was observed in IDE8 but not in HAE/CTVM9, suggesting that the cells from different tick species may have different susceptibility or infection efficiency to KSIV (Figure 2A, bottom panel). In addition, KSIV infection was not observed in the mosquito cell line C6/36 (Figure 2A, bottom panel).

Furthermore, KSIV could replicate efficiently to generate progeny viruses from SW-13, SH-SY5Y, and BHK-21 cells. The virus titer in culture supernatants reached a peak at 24 h p.i. for BHK-21 (4.97 ± 1.13 × 10^8^ TCID_50/_mL) and SW-13 (2.42 ± 1.42 × 10^8^ TCID_50/_mL), while the virus titer was increasing for SH-SY5Y (2.20 ± 0.22 × 10^8^ TCID_50/_mL) until 48 h p.i (Figure 2B).

### Karshi Virus Causes Fatal Encephalitis in Suckling Mice

**FIGURE 3:**
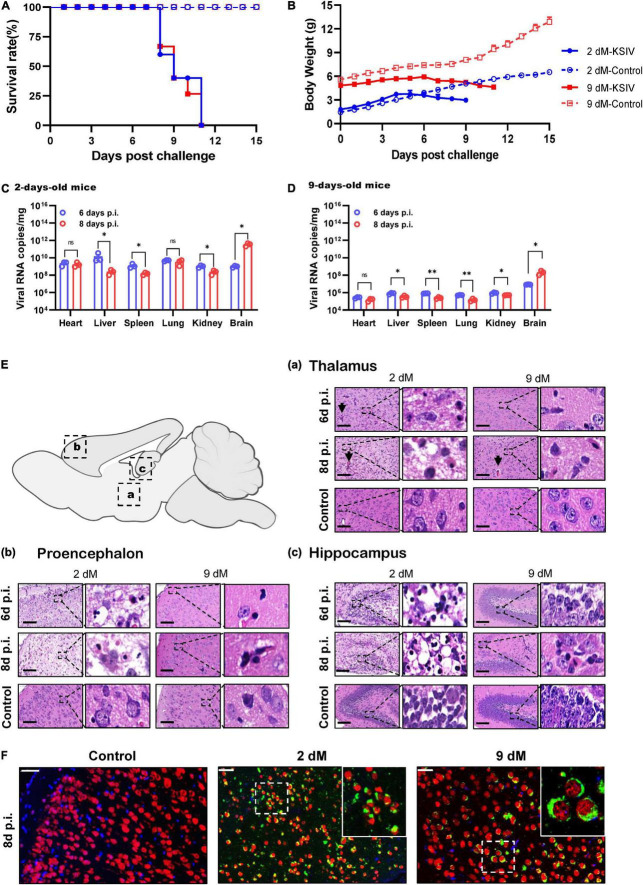
The pathogenicity of KSIV infection in 2- and 9-day-old suckling C57BL/6 mice. The 2- and 9-day-old suckling C57BL/6 mice (2- and 9-dM) were inoculated with KSIV intracranially (1 × 10^4^ PFU) and intraperitoneally (2 × 10^4^ PFU) and monitored for clinical symptoms and mortality over 15 days. **(A)** Survival analysis of mice infected with KSIV. **(B)** Bodyweight changes of mice infected with KSIV. Mice were inoculated with KSIV and different tissues were collected at 6- and 8-days post inoculation (*n* = 3/group) to enable comparisons of virus loads between the 2-day-old **(C)** and the 9-day-old **(D)** infected mice groups. For each time point, the measured values are the average of three mice. Error bars represent standard deviations. Data shown are pooled from three independent experiments. n.s. indicates no significant difference, *p < 0.05, ^**^p < 0.01. **(E)** H&E staining of brains from 2- to 9-day-old KSIV-infected and uninfected suckling mice. At 6- and 8-days post inoculation, mice were euthanized, and brains were H&E stained. Representative images of the brain from **(a)** thalamus, **(b)** prosencephalon, and **(c)** hippocampus. Scale bars represent 100 μm. The enlarged images of interest show details demonstrating inflammation and neuronal necrosis. **(F)** Double immunofluorescence staining was performed on mouse brain sections from 2- to 9-day-old mice 8 days after KSIV infection, and on uninfected mice. The mature neurons were marked with red fluorescence (Alexa Fluor 555), KSIV antigens were stained with green fluorescence (Alexa Fluor 488), and the nuclei were stained with Hoechst 33258.

In an attempt to investigate KSIV pathogenesis, 6-week-old C57BL/6 mice were inoculated with KSIV. However, these mice did not subsequently display any sign of illness, nor any effect on body weight (data not shown). Then, 2- and 9-day-old suckling mice were inoculated with KSIV (Figure 3). The 2-day-old mice showed signs of neurological symptoms, such as drowsiness, stroke and paralysis 3 days after infection (D3), and death occurred on D7. The 9-day-old mice displayed onset of illness from D5, and death occurred on D8. None of these mice survived the KSIV infection (Figure 3A), and their body weight decreased after onset of illness (Figure 3B).

Karshi virus infection in the mouse tissues was determined on D6 and D8. The 2-day-old mice had higher viral loads in all tested tissues than the 9-day-old mice (Figures 3C,D). For the 2-day-old mice, viral loads significantly decreased in the liver, spleen, and kidney from D6 to D8 (*p* < 0.05), while the decrease was not significant in the heart and lung (Figure 3C). In 9-day-old mice, viral loads significantly decreased in the liver, spleen, lung, and kidney from D6 to D8 (*p* < 0.05 or *p* < 0.01), while the decrease in the heart was not significant (Figure 3D). In contrast to the decreased viral loads in these tissues, a significant increase in virus load was observed in the brain from both 2- and 9-day-old mice (*p* < 0.05), suggesting KSIV proliferation in the brain of suckling mice (Figures 3C,D).

Pathological lesions in the brains of diseased suckling mice were characterized by H&E staining. The 2-day-old mice with KSIV infection developed herniation in a large area of the thalamus on D6 and D8, which showed severe tissue oedema, and nuclear pyknosis in neuronal nuclei. The 9-day-old mice also developed brain herniation in restricted regions of the thalamus on D8; however, they had slight brain tissue oedema and inflammatory infiltration (Figure 3Ea). The prosencephalon had severe brain tissue porosity, oedema, pyknosis, and necrosis of neuronal nuclei in 2-day-old mice on D6, and the lesion deteriorated further on D8. However, despite inflammatory cell infiltration being observed in prosencephalon for the 9-day-old mice, the lesions seemed to be less severe than those in the 2-day-old mice (Figure 3Eb). In the hippocampus, inflammatory cell infiltration and necrosis of nerve cells were observed to be more severe in the 2-day-old mice than the 9-day-old mice, and the lesions further deteriorated from D6 to D8 (Figure 3Ec). Generally, the 2-day-old mice had more severe pathological changes due to KSIV infection in the thalamus, forebrain, and hippocampus of the brain than the 9-day-old mice. Furthermore, the results of IFA assays with the mouse brain sections revealed KSIV infection in several neurons in brain tissues of the 2- and 9-day-old mices on D8, suggesting that neurons are the targets of KSIV infection *in vivo* (Figure 3F).

### Molecular Prevalence of Karshi Virus Among Ticks and Seroprevalence Among Animals in the Field Suggesting Karshi Virus Spillover From Ticks to Animal Hosts

**FIGURE 4:**
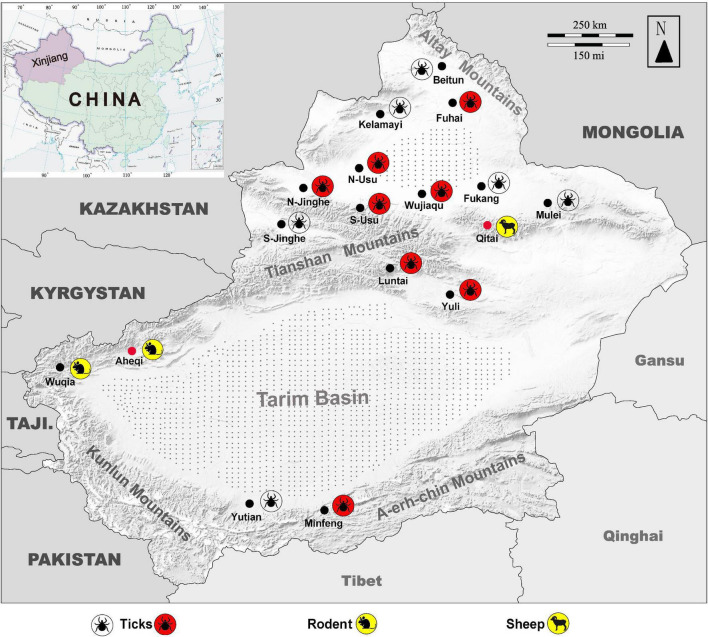
Map of Xingjiang Region showing locations where KSIV infection was detected. Molecular, epidemiological, and serological investigations were conducted on ticks and field-collected animal sera from Xinjiang, revealing that the animals had serological exposure to tick-borne encephalitis virus (TBEV) as well as to KSIV. The map of Xinjiang, China, shows tick sampling locations and molecular epidemiology of KSIV, and anti-KSIV and anti-TBEV antibody detection in animal sera. The silhouette of a tick with a red background represents a sampling location where KSIV nucleic acid was detected, and the silhouette of a transparent tick represents a sampling location where KSIV was not detected. The silhouettes of sheep and rats on a yellow background represent the sampling locations (black dots) where KSIV-antibodies were detected, while the locations where the animal serum samples also had neutralizing antibodies to TBEV are indicated by red dots. Gray stippling indicates arid desert areas. S-Usu, South of Usu City; N-Usu, North of Usu City; S-Jinghe, South area of Jinghe County; N-Jinghe, North of Jinghe County.

KSIV prevalence was investigated among 363 groups of ticks, of which 18 groups were identified as KSIV RNA positive (the minimum infection rate was 4.96%) (Table 1 and Figure 4). Two of the seven *Hy. detritum* tick groups from Fuhai County were positive for KSIV RNA, resulting in a high minimum infection rate of 28.57%. Among *Hy. asiaticum* ticks, KSIV RNA was detected in 10 (8.77%) of the 114 groups, of which the ticks from Luntai had the highest minimum infection rate (two groups, 22.22%), followed by those from Wujiaqu (three groups, 21.43%), Minfeng (two groups, 14.29%), Yuli (one group, 7.15%), and North of Usu (two groups, 6.25%). In contrast, KSIV RNA was not detected among *Hy. asiaticum* ticks from Yutian, Karamay, Beitun, Mulei, or Fukang. Among *D. nuttalli* ticks, 6 of the 242 groups (the minimum infection rate was 2.48%) were positive for KSIV RNA, including two groups from North of Jinghe (5.26%) and four groups from South of Usu (3.92%), while among the 102 groups from the South of Jinghe, KSIV was not detected (Table 1). The partial sequences of NS5 (302 nt) were used to construct a phylogenetic tree, which showed that all the KSIV sequences in Xinjiang clustered together with the new isolate KSIV-WJQ16209 (Supplementary Figure 2).

**TABLE 1:** Prevalence of Karshi virus (KSIV) among groups of *Hyalomma detritum*, *Hyalomma asiaticum*, and *Dermacentor nuttali* ticks collected from vegetation in Xinjiang.

Tick species	Location	Ticks (*n*)	Tick groups	KSIV RNA-positive groups	Minimum infection rate (%)
*Hyalomma detritum*	Fuhai County	326	7	2	28.57
Subtotal		326	7	2	28.57
*Hyalomma asiaticum*	Luntai County	750	9	2	22.22
	Wujiaqu City	1,102	14	3	21.43
	Minfeng County	1,500	14	2	14.29
	Yuli County	1,100	14	1	7.15
	North of Usu City	2,300	32	2	6.25
	Yutian County	103	2	0	0
	Karamay City	783	11	0	0
	Beitun County	70	1	0	0
	Mulei County	560	8	0	0
	Fukang City	473	9	0	0
Subtotal		8,741	114	10	8.77
*Dermacentor nuttalli*	North of Jinghe County	3,700	38	2	5.26
	South of Usu City	9,245	102	4	3.92
	South of Jinghe County	9,530	102	0	0
Subtotal		22,475	242	6	2.48
**Total**		**31,542**	**363**	**18**	**4.96**

Subsequently, KSIV seroprevalence was investigated among domestic (sheep) and wild (marmot, great gerbil and long-tailed ground squirrel) animals (Table 2). Of the serum samples from 329 sheep, only eight (2.43%) samples were anti-KSIV antibody-positive as evidenced by IFA. However, these eight samples were not identified as positive either by western blot, which was performed using the linearized viral proteins from purified KSIV particles, or by LIPS assays, which were conducted using the KSIV E-DIII. This difference could have resulted from using different antigens or from different sensitivities in detecting antibodies by these methods. Nevertheless, one of the eight IFA-positive samples from sheep showed neutralizing activity against KSIV (0.54% of the total number of sheep tested), resulting in a neutralizing titer of 2^6^, which confirmed that KSIV infection had occurred among sheep in Qitai in 2014 (Table 2). Of the 1,098 serum samples tested from wild animals, 28 samples (2.56%) were antibody positive by IFA, among which 10 samples (0.91%) were confirmed as having antibody responses to linearized KSIV antigens by western blot and the E-DIII of KSIV by LIPS assays, respectively (Table 2). Furthermore, seven samples (0.64%) had neutralizing activity against KSIV with the titers ranging from 2^4^ to 2^6^. All the 28 antibody-positive samples were derived from marmots, including 15 collected from Aheqi in 2006 and 13 from Wujiaqu in 2015 (Table 2), whereas none of the tested samples from great gerbil or long-tailed ground squirrel were positive. The results demonstrated evidence of serological exposure to KSIV among marmots as early as 2006 in Xinjiang.

**TABLE 2:** Seroprevalence of antibodies to KSIV among domestic and wild animals in Xinjiang.

Animal	Year	Location	Serum samples	Positive sample number and rates, *n* (%)			VNT number, rates and titer *n*, (%, titer)
				IFA	WB	LIPS	
**Domestic animals**							
Sheep	2014	Qitai County	183	8 (4.37)	0	0	1 (0.54, 2^4^)
		Yuepuhu County	72	0	0	0	0
	2015	Shawan County	74	0	0	0	0
Subtotal			329	8 (2.43)	0	0	1 (0.30, 2^4^)
**Wild animals**							
Marmot	2006	Aheqi County	308	15 (4.87)	2 (0.65)	10 (3.3)	7 (0.23, 2^4^–2^6^)
	2014	Wenquan County	61	0	0	0	0
	2015	Wuqia County	306	13 (4.25)	8 (2.61)	0	0
Great gerbil	2008	Hetian County	59	0	0	0	0
Long-tailed ground squirrel	2014	Yiwu County	364	0	0	0	0
Subtotal			1098	28 (2.56)	10 (0.91)	10 (0.91)	7 (0.64, 2^4^–2^6^)
**Total**			1427	36 (2.52)	10 (0.07)	10 (0.07)	8 (0.06, 2^4^–2^6^)

*IFA, immunofluorescence assays; WB, western blot assays; LIPS, luciferase immunoprecipitation system detection assays; VNT, virus neutralization test assays.*

### Karshi Virus Has Serological Cross-Reactivity With Tick-Borne Encephalitis Virus

The KSIV E protein (KSIV-E) shares 78.3% amino acid sequence identity with TBEV E protein (TBEV-E) (Supplementary Table 1). KSIV-E may have protein structural properties similar to TBEV-E according to the sequence alignment and structure prediction. Like TBEV-E, it has the conserved flavivirus fusion loop “DRGW” and could be divided into four functional domains I, II, III, and IV (Supplementary Figure 3A). It has six disulfide bridges linked by cysteines at conserved positions. The secondary structure of KSIV-E contains 31 β-sheets, 7 η-helices, and 7 α-helices, which is very similar to that of TBEV-E which contains 31 β-sheets, 7 η-helices, and 8 α-helices (Supplementary Figure 3B). Moreover, KSIV-E has a predicted 3D structure similar to that of TBEV (root mean square deviation: 0.225 Å) (Supplementary Figure 3C). All these findings suggested that KSIV-E and TBEV-E have a similar mode of function as well as protein antigenicity, further indicating that there may be a serological association between KSIV and TBEV.

**FIGURE 5:**
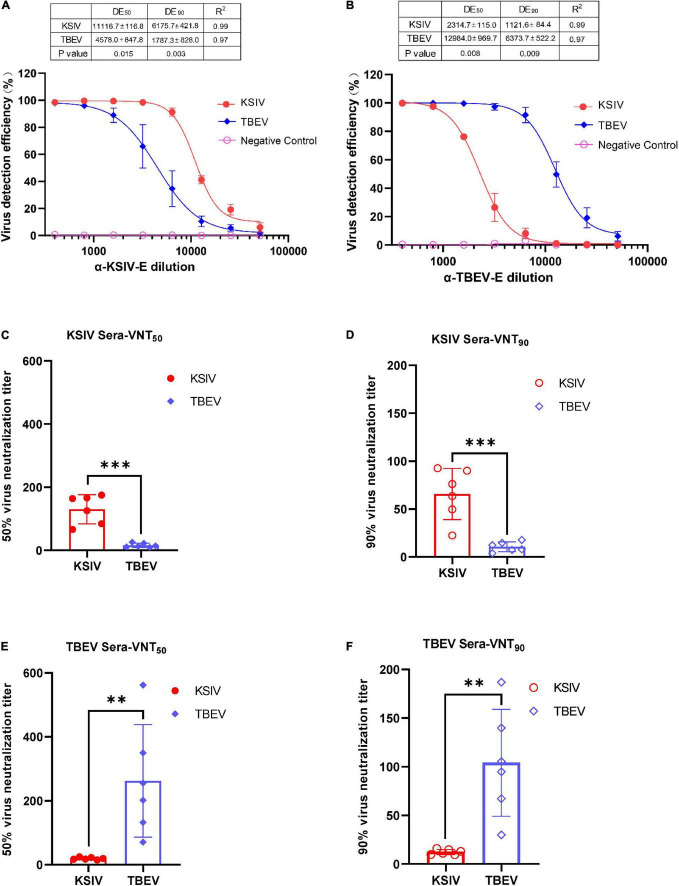
Karshi virus has serological cross-reactivity with TBEV. The determination of KSIV and TBEV serological cross-reaction and neutralization was based on the immunofluorescence test. Percentages of KSIV-infected/TBEV-infected cells were obtained and normalized with the Operetta High-Content Imaging System (PerkinElmer). KSIV and TBEV serological cross-reaction test. The efficiencies of the lab-prepared polyclonal antibodiesα-KSIV-E **(A)** and α-TBEV-E **(B)** reacting to the KSIV- and TBEV-infected cells were evaluated using GraphPad Prism software (Version 8) least square fitting method to determine the detection efficiency curve. The KSIV-infected mouse serum dilution was determined as an antibody titer that inhibited 50% **(C)** and 90% **(D)** of KSIV and TBEV infection. The TBEV-infected mouse serum dilution was determined as an antibody titer that inhibited 50% **(E)** and 90% **(F)** of KSIV and TBEV infection. The data were shown as means ± SD. ^**^p < 0.01, ^***^p < 0.001.

As expected, the lab-prepared polyclonal antibody, α-KSIV-E, could efficiently recognize KSIV antigen, resulting in a DE_50_ of 11,116.7 ± 116.8 and DE_90_ of 6,175.7 ± 421.8, while its recognition of TBEV antigen was significantly reduced (DE_50_: 4,578.0 ± 847.8, *p* < 0.05; DE_90_: 1,787.3 ± 828.0, *p* < 0.001; Figure 5A). Moreover, antibody α-TBEV-E could recognize both TBEV and KSIV antigens, but with efficiency to KSIV (DE_50_ and DE_90_: 2,314.7 ± 115.0 and 1,121.6 ± 84.4) significantly lower than to TBEV (DE_50_ and DE_90_: 12,984.0 ± 969.7 and 6,373.7 ± 522.2) (*p* < 0.001; Figure 5B). Therefore, the anti-serum prepared in the lab against KSIV-E cross-reacted with TBEV, and vice versa, which confirmed the serological relationship between these two viruses.

The serological cross-reactivity between KSIV and TBEV was further demonstrated by determining neutralizing activities of the serum samples from KSIV- or TBEV-infected mice. The serum samples from KSIV-infected mice could effectively neutralize KSIV, resulting in VNT_50_ of 130.1 ± 18.9 and VNT_90_ of 65.8 ± 10.9. These serum samples could also neutralize TBEV infection, resulting in significantly reduced titers of VNT_50_ (15.8 ± 2.8) and VNT_90_ (10.64 ± 2.1) (*p* < 0.001; Figures 5C,D). This was similar to the serum samples from TBEV-infected mice, which could effectively neutralize TBEV (VNT_50_ = 262.0 ± 71.9; VNT_90_ = 104.0 ± 22.4) and could cross-neutralize KSIV with reduced efficiency (VNT_50_ = 19.3 ± 1.5; VNT_90_ = 12.3 ± 1.1) (*p* < 0.05; Figures 5E,F).

### Animals in Xinjiang Had Serological Exposure to Tick-Borne Encephalitis Virus as Well as to Karshi Virus

Based on the above results, we speculated that the KSIV-antibody-positive serum samples from animals might cross-react with TBEV. The thirty-six KSIV antibody-positive serum samples and three randomly selected negative serum samples were used to detect antibody reaction with TBEV by IFAs, western blot and LIPS assays as well as by virus neutralization assays to evaluate cross-neutralization of TBEV. The three KSIV-negative samples were still negative with TBEV (Supplementary Figure 4). Twenty-five (64.9%) of the 36 KSIV-antibody-positive samples had antibody responses to TBEV by IFA, among which 14 samples (38.9%) were confirmed as having antibody responses to linearized TBEV antigens by western blot and seven (19.4%) having a response to the E-DIII of TBEV by LIPS assays, respectively (Table 3 and Supplementary Figure 4). Furthermore, eight (32%) of the twenty-five TBEV antibody-positive serum samples had neutralizing activity against TBEV, resulting in titers ranging from 2^3^ to 2^8^; these samples comprised four from marmot in Aheqi and four from sheep in Qitai (Table 3 and Supplementary Table 2). These results indicated that infection with KSIV or TBEV, or with both viruses, could have occurred in these animals.

**TABLE 3:** Antibody response against tick-borne encephalitis virus among Karshi virus-IFA positive animal serum samples.

Species	Location	No.	Positive sample number and rates, *n* (%)			
			IFAs	WB	LIPS	By-Neutralization
Marmot	Wuqia County	13	10 (76.9)	9 (69.2)	1 (7.7)	0
	Aheqi County	15	9 (60.0)	3 (20.0)	3 (20.0)	4 (26.7)
Sheep	Qitai County	8	6 (75.0)	2 (25.0)	3 (37.5)	4 (50.0)
**Total**		**36**	**25 (69.4)**	**14 (38.9)**	**7 (19.4)**	**8 (32.0)**

*IFA, immunofluorescence assays; WB, western blot assays; LIPS, luciferase immunoprecipitation system detection assays.*

Based on the detailed results of detecting antibody reaction to TBEV among the 36 KSIV antibody-positive animal serum samples as listed in Supplementary Table 2, we proposed that at least five samples (one from marmot and four from sheep) were more likely to have been exposed to TBEV than to KSIV. First, regarding the neutralization activity against TBEV of the five samples, one (11–107) had a neutralizing titer to TBEV higher than to KSIV, and four (6–59, 7–334, 7–168, and 7–177) had neutralizing activity against TBEV but not against KSIV. Second, regarding the fold changes of the light units normalized to the cut-off value by LIPS, which represented the levels of an antibody reacting to E-DIII of the respective virus, three of the five samples (6–59, 7–334, and 7–168) were antibody-positive for TBEV but negative for KSIV. One (11–107) had a two-fold higher level of antibody reaction to TBEV than to KSIV. One other sample (7–177) from the five did not show a response to TBEV E-D III, probably due to the limitation of LIPS assay to detect antibody specific to epitopes more than those in domain III. Nevertheless, its positive reaction to TBEV antigen by IFA and neutralization activity against TBEV still suggested this animal was previously exposed to TBEV. Moreover, we proposed that four other samples (three from marmot and one from sheep) were more likely to have exposure to KSIV rather than to TBEV, as they (11–260, 10–191, 10–264, and 6–132) had neutralization specifically against KSIV but not TBEV, and were antibody-positive to KSIV-E rather than to TBEV-E. Furthermore, three samples had neutralization activity against both viruses, including one sample (11–199) with higher neutralization titer to KSIV than to TBEV, and two others (11–189 and 10–258) showing identical neutralizing titers to both viruses. For the sample 11–199, although its reaction to KSIV and TBEV was negative by LIPS, its antibody responses to both viruses were confirmed by IFAs and western blot assays. Sample 11–189 showed an antibody response to TBEV slightly higher than to KSIV, while sample 10–258 had an antibody response to KSIV but not to TBEV by LIPS assays. Their serological reactions to KSIV and TBEV antigens were both confirmed by IFAs but not by western blot assays. Therefore, we speculated that these three samples were likely to have co-exposure to KSIV and TBEV, or that due to the close serological correlation between KSIV and TBEV, the two samples have an infection with one of the two viruses and showed effective antibody cross-reaction with the other.

## Discussion

Karshi virus was first isolated from *Ornithodoros papillipes* ticks collected in Uzbekistan in 1972 (Lvov et al., 1976), and subsequently from *Hy. asiaticum* ticks collected in the north of Central Asia (Alma-Ata region of the Kazakh Soviet Socialist Republic) in 1976 and from *Ornithodoros caniceps* collected in Turkmenistan in 1978 (Khutoretskaya et al., 1985). In the present study, a strain of KSIV was isolated from *Hy. asiaticum* ticks in Xinjiang, Northwestern China. Investigation of KSIV prevalence revealed that *Hy. detritum*, *Hy. asiaticum* and *D. nuttalli* ticks collected in Xinjiang could be the natural reservoirs of this virus, with the highest minimum infection rate (28.57%) among the *Hy. detritum* groups, followed by the *Hy. asiaticum* and *D. nuttalli* groups. Although the rates could be affected by geographically limited sampling of ticks, which was performed mostly around the Tianshan Mountainous regions, the resulting minimum infection rates of 4.96% in total still suggested the substantial prevalence of KSIV in Xinjiang, especially around the pasture and farming areas relying on irrigation from the Tianshan Mountains. Moreover, KSIV was proved to be transmitted by *Ornithodoros* ticks and was able to replicate in ixodid ticks in laboratory experiments (Lvov et al., 1976; Aristova et al., 1986; Turell et al., 2004). Our study showed that KSIV could infect the *I. scapularis* cell line IDE8; however, KSIV infection was not observed in the *Hy. anatolicum* cell line HAE/CTVM9. This could be because KSIV had different infection and replication efficiencies in the *Hyalomma* and *Ixodes* cells at the tested time point (48 h p.i.). In a comparative *in vitro* study, levels of infective TBEV particles produced between 24 and 48 h.p.i. increased in *Ixodes* spp. cell lines, but decreased in HAE/CTVM9 cultures (Růzek et al., 2008). Alternatively, the HAE/CTVM9 cells may have been unable to support production of KSIV E protein or were refractory to infection. The results of KSIV detection in *Hy. detritum*, *Hy. asiaticum*, and *D. nuttalli* ticks and KSIV infection in the *I. scapularis* cell line, together with previous studies on KSIV isolation, transmission, and replication in other tick species (Lvov et al., 1976; Khutoretskaya et al., 1985; Terskikh et al., 1989; L’Vov et al., 1994; Turell et al., 2004, 2008; Turell, 2015), suggested that multiple tick species may harbor KSIV, which indicated the importance of performing a survey on KSIV molecular prevalence among more tick species from wider geographic areas.

Tick-borne encephalitis virus is a notorious viral pathogen, which causes acute encephalitis accompanied by myelitis, and may be fatal to humans (Lindquist and Vapalahti, 2008). Experimental animals including rodents, dogs, horses, monkeys, cows, goats, and sheep were susceptible to TBEV infection (Nalca et al., 2003; Klaus et al., 2014). TBEV infection with European, Siberian, and Far Eastern subtypes resulted in mild to severe symptoms in experimental animals, suggesting that different viral strains may have differential pathogenicity (Nalca et al., 2003). Nevertheless, these the experimental animals susceptible to TBEV exhibited neuronal damage, necrosis or encephalitis associated with inflammatory cell infiltration. Due to its close relationship to TBEV, KSIV may be an emerging tick-borne viral pathogen, which should be evaluated for its potential threats to infect different hosts. A previous study reported that KSIV could infect animals such as white mice, Syrian hamsters and green monkeys, but lacked details about the pathological lesions and responses of these animals (Terskikh et al., 1989). Moreover, it is unknown whether KSIV could infect and cause disease in humans. The current study showed that KSIV could infect cell lines derived from different tissues of humans and mammals including monkeys, hamsters, sheep, and dogs. The efficient replication of KSIV in the human kidney and neural cell lines, as well as the hamster cell line, suggested that KSIV has the potential to infect humans and other animals. We attempted to investigate KSIV pathogenesis in adult C57BL/6 mice in this study. Although these mice did not show any symptoms, detection of neutralizing antibody to KSIV in serum samples from these mice suggested the occurrence of transient infection and humoral immunity caused by KSIV infection. As expected, KSIV infection resulted in fatal disease in the suckling mice, which are supposed to have a naïve or developing immune system. Further investigation of KSIV infection in different species of experimental animals would promote our understanding of its pathogenesis and reveal whether its pathology is similar to that of TBEV infection.

Karshi virus RNA was detected in mouse tissues collected during the acute infection phase. While the viral loads decreased or were maintained in most tested tissues from D6 to D8 after KSIV infection, the viral load in the brain significantly increased. Moreover, severe lesions were observed in the thalamus, prosencephalon, and hippocampus of the mouse brain, and KSIV antigen expression was detected in the neurons. The results showed that KSIV could infect and replicate in the brain of mice with developing immune systems, and result in encephalitis, which suggested that KSIV is pathogenic by causing lesions in the brain and infecting neurons in the same way as TBEV. Investigation of serum samples from domestic and wild animals further revealed serological evidence of KSIV exposure among sheep from Qitai and marmots from Aheqi and Wuqia. This suggested that KSIV spillover had occurred to domestic and wild animals.

The serological correlation between KSIV and TBEV was confirmed by detecting the efficiency of laboratory-prepared KSIV antibodies in reacting with TBEV antigen and neutralizing TBEV infection, and vice versa. Therefore, we speculated that the KSIV antibody-positive animal serum samples identified in this study might include those having serological exposure to KSIV and showing cross-reaction to TBEV, and those having serological exposure to TBEV and also showing cross-reaction to KSIV. As expected, the serological tests with 36 KSIV antibody-positive animal serum samples confirmed that, at most, 25 samples had antibody responses to TBEV. By carefully analyzing the detailed results, we found that at least five among the 36 samples (13.9%) were more likely to have TBEV exposure and showed serological cross-reaction to KSIV. Four samples (11.1%) had infection with KSIV and showed cross-reaction to TBEV without cross-neutralization against TBEV. Three of the 36 sampled animals (8.3%) might have had co-exposure to both TBEV and KSIV or might have had an infection with one of these two viruses and showed strong cross-reactivity to the other. Our results suggested that both KSIV and TBEV infections have occurred among animals in Xinjiang.

Since the 1990s, the areas close to Tianshan Mountains and Altay Mountains have been considered epidemic foci of forest encephalitis in Northwestern China (Bi et al., 1997). High TBEV seroprevalence among humans and animals including marmots and sheep was also reported from there (Xie et al., 1991). The rate of TBEV seroprevalence in humans was 15.9% from 2011 to 2015, and in marmots from the Tianshan Mountains, this rate reached up to 75%, which is in contrast to the very few numbers of TBEV-infected human cases that occurred throughout the whole province (Men et al., 2021). Here, we presented the locations in a map to show where tick groups were detected positive for KSIV RNA, and where animals had positive antibody responses to KSIV and/or TBEV (Figure 4). While ticks collected from areas close to Tianshan Mountains (Usu, Jinghe, Wujiaqu, Luntai, and Yuli) as well as from Altay Mountains (Fuhai) and Kunlun Mountains (Minfeng) were KSIV-positive, a serological response to KSIV was detected among animals in the areas along Tianshan Mountains (Wuqia, Aheqi, and Qitai) where antibody response to TBEV in animals was also found (Figure 4). Therefore, according to the results of our study, we speculated that there could be a misinterpretation of the high TBEV seroprevalence. First, the rates may have included human cases and animals having KSIV infection or exposure due to the close serological relationship between these two viruses. Second, the regions of high KSIV prevalence among ticks, including the counties around Tianshan Mountains as well as Altay Mountains, merged with the natural foci of TBEV. Therefore, we suggest that it is necessary to perform follow-up surveys on both TBEV and KSIV by detecting viral RNA among human patients, domestic and wild animals and ticks, to further investigate in depth the substantial prevalence of both viruses among vectors and hosts. The results also raised the need to develop specific serological detection methods to distinguish KSIV infection from TBEV, which may promote understanding of the seroprevalence of KSIV and TBEV among humans and animals and further improve diagnosis and therapeutic strategies regarding infectious diseases related to these two viruses.

Very few studies performed surveys of tick species parasitic on animals in Xinjiang; nevertheless, it was found that 45.4% of juvenile gray marmots in Shawan County from the Tianshan Mountains carried *I. persulcatus* (Xie et al., 1991), and that in Qitai County, the tick species carried by sheep included *Hy. asiaticum*, *D. nuttalli*, *Hy. anatolicum*, *Dermacentor pavlovsky*, and *Rhipicephalus sanguineus* (Wang Y. et al., 2015; Sheng et al., 2019; Li et al., 2020). These reports suggested that ticks, during their life cycles, had substantial contact with marmots and sheep, which indicated the potential for virus transmission between the tick vectors and animal hosts. In the present study, there were limitations on time and locations of sample collection, and the locations of KSIV-positive ticks were not consistent with those of KSIV antibody-positive animals, which may not accurately reflect the possibility of direct transmission of KSIV from ticks to animal hosts. Since we found serological evidence of the occurrence of KSIV exposure in marmots and sheep, further investigation of ticks and animal serum samples collected from the same locations in more regions in Northwestern China may further clarify the threat of KSIV transmission from ticks to animal hosts. One other limitation of the present study is that serological exposure of KSIV or KSIV infection incidence among humans were not characterized due to the lack of human samples. Nevertheless, our results of serological correlations between KSIV and TBEV, together with the previous reports on high TBEV seroprevalence among humans in Xinjiang, indicated that KSIV might be prevalent in the areas reporting TBEV antibody-positive humans.

In summary, this study isolated a new KSIV strain and reported the prevalence of KSIV among ticks in Xinjiang, Northwestern China. KSIV was able to infect various cell lines from humans, animals and ticks, caused encephalitis in experimental mice (with pathogenesis similar to TBEV) and had low seroprevalence among marmots and sheep. All these findings suggested that KSIV is a potential pathogen with the ability to spill over from ticks and animal hosts to humans. This study further evaluated the serological cross-reactivity between KSIV and TBEV and found that KSIV and TBEV infection have both occurred among animals, which indicated that there has been misinterpretation of the high seroprevalence of TBEV among humans and animals in Xinjiang. The results of this study have improved our knowledge of the etiology and epidemiology of tick-borne flavivirus pathogens in Xinjiang. Moreover, the findings suggested the necessity to perform an extensive investigation of KSIV prevalence and incidence in Northwestern China, which may enhance our preparation to provide medical care against emerging diseases caused by tick-borne viral pathogens such as KSIV.

## Data Availability Statement

The datasets presented in this study can be found in online repositories. The names of the repository/repositories and accession number(s) can be found below: http://db.cngb.org/cnsa/project/CNP0002610/reviewlink/, CNP0002610.

## Ethics Statement

The animal study was reviewed and approved by the ethics committee of Wuhan Institute of Virology, Chinese Academy of Sciences.

## Author Contributions

YuZ, SSu, ZS, and AM collected the tick samples and performed the morphological classification of ticks. YaZ, ST, and SSh grouped the ticks and prepared the homogenates. AM extracted RNA from ticks. LB-S established the *Hyalomma* tick cells and provided suggestions on virus infection in tick cells. SSh performed the molecular classification of ticks and directed RNAseq analyses. JW and SSh analyzed the RNAseq data. SSu and YuZ collected the animal serum samples. FD and SSh conceived the study. YB conducted the experiments and analyzed the data. YB and SSh wrote the manuscript. SSu, SSh, LB-S, and FD reviewed and finalized the manuscript. All authors contributed to the article and approved the submitted version.

## Conflict of Interest

The authors declare that the research was conducted in the absence of any commercial or financial relationships that could be construed as a potential conflict of interest.

## Publisher’s Note

All claims expressed in this article are solely those of the authors and do not necessarily represent those of their affiliated organizations, or those of the publisher, the editors and the reviewers. Any product that may be evaluated in this article, or claim that may be made by its manufacturer, is not guaranteed or endorsed by the publisher.

## Footnotes

1
http://db.cngb.org/cnsa/project/CNP0002610/reviewlink/

2
https://espript.ibcp.fr/ESPript/ESPript/

3
http://www.nhc.gov.cn/wjw/gfxwj/201304/64601962954745c1929e814462d0746c.shtml
