# Supplementary material for: Discovery of Tick-Borne Karshi Virus Implies Misinterpretation of the Tick-Borne Encephalitis Virus Seroprevalence in Northwest China
Source: Front Microbiol. 2022 May 3;13:872067. doi: 10.3389/fmicb.2022.872067 (PMC9173002; doi:10.3389/fmicb.2022.872067)
Supplement: Supplementary file 1 [file Data_Sheet_1.docx]

## Supplementary Material

# Supplementary Methods

## Viruses and cell lines

Mammalian and mosquito cell lines used in this study were obtained from the American Type Culture Collection (ATCC, Manassas, VA, USA), namely SH-SY5Y (human neuroblastoma cells; ATCC number: CRL-2266), U-87 MG (human glioma cells; ATCC number: HTB-14), HEK293 and 293T (human embryonic kidney cells; ATCC number: CRL1573 and CRL-3216), SW-13 (human adrenocortical carcinoma cells; ATCC number: CCL-105), HepG2 (human liver cells; ATCC number: HB-8065^TM^), BHK-21 (baby hamster kidney cells; ATCC number: CCL-10), Vero (African green monkey cells; ATCC number: CCL-81), and DH82 (canine macrophage cells; ATCC number: CRL-10389) cells, which are grown in Dulbecco’s modified Eagle’s medium (DMEM; Sigma, St. Louis, MO, USA) supplemented with 10% fetal bovine serum (Gibco, Grand Island, NY, USA), and MDBK (bovine kidney cells; ATCC number: CCL-22), MDOK (Madin–Darby ovine kidney cells; ATCC number: CRL-1633), C6/36 (mosquito cells were derived from *Aedes albopictus*; ATCC number: CRL-1660), and were grown in Minimum Essential Medium (MEM; Sigma) supplemented with 10% FBS. Two tick cell lines, IDE8 derived from *Ixodes scapularis* and HAE/CTVM9 derived from *Hyalomma anatolicum*, were obtained from the Tick Cell Biobank, University of Liverpool, and were cultured as previously described ([Bell-Sakyi, 1991](#_ENREF_1); [Munderloh et al., 1994](#_ENREF_4)).

The TBEV strain NM-Tick-2020 was isolated from ticks from Inner Mongolia, China, and deposited in the National Virus Resource Center (CSTR: 16533.06.; IVCAS 6.7508), Wuhan Institute of Virology, Chinese Academy of Sciences. Virus titres were determined using BHK-21 cells by the microtitration method, and they were expressed as the 50% tissue culture infective dose (TCID_50_) according to the Reed-Muench method.

## Preparation of the KSIV and TBEV E protein polyclonal antibodies (α-KSIV-E and α-TBEV-E)

KSIV and TBEV RNA was extracted using TRIzol (Invitrogen, Carlsbad, USA) and reverse-transcribed into cDNA using M-MLV reverse transcriptase (Takara, Kusatsu, Japan). To construct prokaryotic expression plasmids of KSIV and TBEV envelope (E) proteins (KSIV-E and TBEV-E), the truncated envelope genes lacking the transmembrane regions were amplified using the viral cDNA as templates by PCR with 2×Rapid Taq Master Mix (Vazyme Biotech, Nanjing, China) with KSIV primers, KSIV-E-F: 5′-CTAGCTAGC(NheI)GTGACAGGAACCCAGGGAACTACCCTG -3′ and KSIV-E-R: 5′-CCCAAGCTT(HindIII)TTACGCCTTCCCGACTGACCCGAAG -3′, and TBEV primers TBEV-E-F: 5′-CGGGCTAGC (NheI)ATTACTGGCACTCAGGGAACCACTCG -3′, and TBEV-E-R: 5′-CCCAAGCTT(HindIII)TTACGCCTTACCAACCGAAGTCAGGAATC -3′.

The PCR products were cloned into the plasmid pET-28a (Novagen, Carlsbad, CA, USA) to generate the expression plasmids pET-28a-KSIV-E and pET-28a-TBEV-E, which were verified by Sanger sequencing. Protein expression, purification, and rabbit immunization were conducted as previously described ([Deng et al., 2007](#_ENREF_3)) to prepare rabbit antiserum α-KSIV-E and α-TBEV-E.

## Analyses of KSIV infectivity, serology and histopathology in mice

Six-week-old female C57BL/6 mice were inoculated with KSIV at 2×10^5^ plaque-forming unit (PFU)/mouse intraperitoneally, and their body weight and survival were monitored continuously for 4 weeks. The surviving mice were sacrificed and serum was collected at the end of the experiment and stored at -80 °C until further examination.

Two-day-old and 9-day-old C57BL/6 suckling mice were inoculated with KSIV by both intraperitoneal (2×10^4^ PFU) and intracerebral (1×10^4^ PFU) routes, while the negative controls received cell culture supernatants from uninfected BHK-21 cells. The mice were monitored over fifteen days for signs of illness, as well as bodyweight changes and survival. On day (D) 6 and D8 after the virus inoculation, three mice in each group were sacrificed, and tissues (heart, liver, spleen, lungs, kidneys, and brain) were collected to measure virus loads by qRT-PCR. Brains were fixed in 4% paraformaldehyde-PBS, and H&E staining was performed to characterize tissue lesions in the thalamus, prosencephalon and hippocampus.

Co-localization by double IFA staining of neuronal nuclei and fluorophores in KSIV-infected cells was performed as previously described ([Sakai et al., 2013](#_ENREF_5)), to visualize KSIV-infected neurons from the brains of 2-day-old and 9-day-old mice 8 days after inoculation. Briefly, Anti-NeuN antibodies (Abcam, Cambridge, UK) were used as primary antibodies and incubated with the secondary antibody Goat pAb to Rb IgG conjugated to Alexa Fluor 555 (Abcam) to stain the mature neurons (red fluorescence). After washing thoroughly with PBS, the α-KSIV-E was used as the primary antibody, and Goat pAb to Rb IgG conjugated to Alexa Fluor 488 (Abcam) was used as the secondary antibody to detect the presence of KSIV antigens as green fluorescence in brain tissues, then the nuclei were stained with Hoechst 33,258 (Beyotime, Shanghai, China). The images were taken using the Pannoramic MIDI system (3DHISTECH, Budapest, Hungary).

## Detection of KSIV-RNA in ticks and animal tissues

To investigate the KSIV prevalence among tick groups, total RNA was extracted from homogenates (300 μL) of each group of ticks using TRIzol (Invitrogen) and transcribed into cDNA. PCR was performed with primers KSIV-NS5-F: 5′- CTGAGYTGGCCCTGGAACTCC -3′ and KSIV-NS5-R: 5′- GCTCCCCTTGGCCGAACCGA -3′ to amplify a partial fragment (302 nt) of the NS5 segment in a 50 μL reaction volume containing 1-3 μL cDNA templates using 2×Rapid Taq Master Mix (Vazyme Biotech) according to the manufacturer’s instructions. The PCR products were confirmed by Sanger sequencing.

Real-time reverse transcription-polymerase chain reaction (qRT-PCR) was performed to detect viral loads in mouse tissues. Total RNA was extracted from tissues collected from three mice in the KSIV-inoculated and control groups. The qRT-PCR was performed with total RNA from tissues as the template and with primers KSIV-NS5-F: 5′- GCATCCAGACCTTCCGTGCTGAG -3′ and KSIV-NS5-R: 5′- GGTCCGGGTCACTCTCTCCAATGTC -3′ to amplify a partial fragment (187 nt) of the NS5 protein using the One-Step SYBR Prime Script PLUS RT-PCR Kit (Takara) according to the manufacturer’s instructions. The viral loads in tissues were expressed as KSIV RNA copies / mg of tissue. Each test was performed in triplicate.

## Immunofluorescence assay (IFA)

All IFA tests were performed following the procedures as previously described, with slight modifications ([Shen et al., 2018](#_ENREF_6)). Briefly, to detect KSIV infection in different cell lines, the cells were fixed and permeabilised at 48 h post-infection and incubated with the in-house prepared polyclonal antibody α-KSIV-E as the primary antibody and goat anti-rabbit IgG H&L-fluorescein isothiocyanate (FITC; Abcam) as the secondary antibody.

To detect the serological cross-reaction between KSIV and TBEV, KSIV- or TBEV-infected BHK-21 cells were fixed and permeabilised, and were further incubated with α-KSIV-E or α-TBEV-E, which were 2-fold serially diluted from 1:400 to 1:51200, and goat anti-rabbit IgG H&L-FITC (Abcam) as the secondary antibody. Cell nuclei were stained with Hoechst 33258 (Beyotime). The numbers of total cells and cells with green fluorescence were calculated and analyzed using Harmony High-Content Imaging and Analysis Software (PerkinElmer, USA).

To detect cross-neutralisation using the antisera from KSIV- or TBEV-infected mice or animals sampled in Xinjiang against each of these two viruses, the BHK-21 cells incubated with the mixture containing antisera and TBEV or KSIV (100 TCID_50_ per test) were fixed and permeabilised at 24 h post-infection, incubated with α-KSIV-E or α-TBEV-E as primary antibody, and then incubated with goat anti-rabbit IgG H&L-FITC (Abcam) as the secondary antibody.

To survey the seroprevalence of KSIV among the animals sampled in Xinjiang and their antibody reaction to TBEV, the KSIV-or TBEV-infected BHK-21 were fixed and permeabilised, and further incubated with diluted (1:100) animal serum as the primary antibody, while α-KSIV-E and α-TBEV-E were used as positive controls. FITC-labelled Protein A/G (Abcam) was used as a secondary antibody. Cell nuclei were stained with Hoechst 33258 (Beyotime).

## Western blot assays

Culture supernatants from the KSIV-or TBEV-infected BHK-21 cells were centrifuged at 3000×*g* for 10 min to remove cell debris and then ultracentrifuged (SW41 rotor; Beckman Fullerton, CA, USA) at 150000 × *g* for 3h to purify the viral particles. The purified viral particles were resuspended in phosphate-buffered saline (PBS) and denatured in 1× SDS sample buffer with 1.25% β-mercaptoethanol (Sigma) at 100°C for 10 min to generate the linearised antigen of viral proteins. Then, SDS-PAGE was performed with the linearised viral proteins, which were further transferred to 0.2 µm nitrocellulose membranes (Millipore, Billerica, MA, USA) using a semidry blot format (Trans-Blot Turbo Transfer System; BioRad). After blocking with Tris-buffered saline (TBS) with skimmed milk powder (5% v/v), the membrane was incubated with animal serum samples (1:50 diluted in PBS), or α-KSIV-E and α-TBEV-E (1:4000 dilution) as a positive control, and horseradish peroxidase (HRP)-conjugated ProteinA/G (Trans-Gen Biotech, Beijing, China) as the secondary antibody.

## Luciferase Immunoprecipitation System (LIPS)

The KSIV- and TBEV-E protein domain III fragments (E-DIII, 336 base pairs, bp) were obtained by PCR and cloned into the expression vector pREN2 (provided by Linfa Wang [Duke University, Singapore] and Peng Zhou [Wuhan Institute of Virology, Chinese Academy of Sciences, China]), which has a dual fluorescence (sea cucumber luciferase and *Renilla* luciferase reporter gene) detection system ([Burbelo et al., 2009](#_ENREF_2)). The two recombinant plasmids were transfected into 293T cells and lysed using *Renilla* luciferase reporter gene experimental cell lysate (RG129M; Beyotime). The luciferase light unit (LU) of each Ruc-viral antigen was measured using a Renilla-Lumi^TM^ Plus Renilla Luciferase Reporter Gene Assay kit (Beyotime), and data were analyzed using Promega GloMax^®^-Read (Promega, Madison, WI, USA).

To detect antibodies recognizing E-DIII to KSIV, the 36 KSIV-IFA-positive serum samples and three randomly-selected KSIV-IFA-negative animal sera were diluted 1:50 with buffer A (50 mM Tris, pH 7.5, 100 mM NaCl, 5 mM MgCl2, 1% Triton X-100). The LU values were measured as described previously ([Uehara et al., 2019](#_ENREF_7)). Similarly, the antibody reaction to TBEV E-DIII was measured by LIPS using the animal serum samples that were positive for TBEV antibody as tested by IFA and three other randomly-selected negative animal serum samples. The polyclonal antibodies α-KSIV-E and α-TBEV-E were used as positive controls for the above assays. The mean value of the LU of all serum samples tested multiplied by three times the SD value was the threshold value.

## Virus Neutralisation assays

Neutralisation assays were carried out using BHK-21 cells to test the neutralizing activity of the animal serum samples against KSIV and TBEV. BHK-21 cells were inoculated onto a 96-well cell plate to 75–85% confluence. The serum samples were inactivated at 56 °C for 30 min. The next day, for each test, serum samples (50 μL) were serially diluted two-fold (from 2-3 to 2-8), mixed with equal volumes of supernatants containing viruses at 100 TCID_50_, and further incubated at 37°C for 1.5 h. The mixture was then added to BHK-21 cells and maintained at 37°C for 24 h. IFAs were performed to visualise viral infection in the cells. The neutralisation titres were expressed as the reciprocal of the dilution which prevented virus infection.

To determine serological cross-neutralisation of serum samples from the KSIV-challenged C57BL/6 mice to TBEV and from the TBEV-challenged mice to KSIV, serum samples from each mouse were serially diluted and incubated with KSIV or TBEV (100 TCID_50_ for each test). Neutralisation assays were performed as described above. IFAs were performed to visualise the virus-infected cells.

KSIV-IFA-positive or TBEV-IFA-positive animal sera were collected in Xinjiang; neutralisation assays were performed with KSIV or TBEV as described above.

## References

Bell-Sakyi, L. (1991). Continuous cell lines from the tick Hyalomma anatolicum anatolicum. *J Parasitol* 77(6)**,** 1006-1008.

Burbelo, P.D., Hoshino, Y., Leahy, H., Krogmann, T., Hornung, R.L., Iadarola, M.J., et al. (2009). Serological diagnosis of human herpes simplex virus type 1 and 2 infections by luciferase immunoprecipitation system assay. *Clin Vaccine Immunol* 16(3)**,** 366-371. doi: 10.1128/cvi.00350-08.

Deng, F., Wang, R., Fang, M., Jiang, Y., Xu, X., Wang, H., et al. (2007). Proteomics analysis of Helicoverpa armigera single nucleocapsid nucleopolyhedrovirus identified two new occlusion-derived virus-associated proteins, HA44 and HA100. *J Virol* 81(17)**,** 9377-9385. doi: 10.1128/jvi.00632-07.

Munderloh, U.G., Liu, Y., Wang, M., Chen, C., and Kurtti, T.J. (1994). Establishment, maintenance and description of cell lines from the tick Ixodes scapularis. *J Parasitol* 80(4)**,** 533-543.

Sakai, K., Nagata, N., Ami, Y., Seki, F., Suzaki, Y., Iwata-Yoshikawa, N., et al. (2013). Lethal canine distemper virus outbreak in cynomolgus monkeys in Japan in 2008. *J Virol* 87(2)**,** 1105-1114. doi: 10.1128/jvi.02419-12.

Shen, S., Duan, X., Wang, B., Zhu, L., Zhang, Y., Zhang, J., et al. (2018). A novel tick-borne phlebovirus, closely related to severe fever with thrombocytopenia syndrome virus and Heartland virus, is a potential pathogen. *Emerg Microbes Infect* 7(1)**,** 95. doi: 10.1038/s41426-018-0093-2.

Uehara, A., Tan, C.W., Mani, S., Chua, K.B., Leo, Y.S., Anderson, D.E., et al. (2019). Serological evidence of human infection by bat orthoreovirus in Singapore. *J Med Virol* 91(4)**,** 707-710. doi: 10.1002/jmv.25355.

# Supplementary Figures and Tables

**
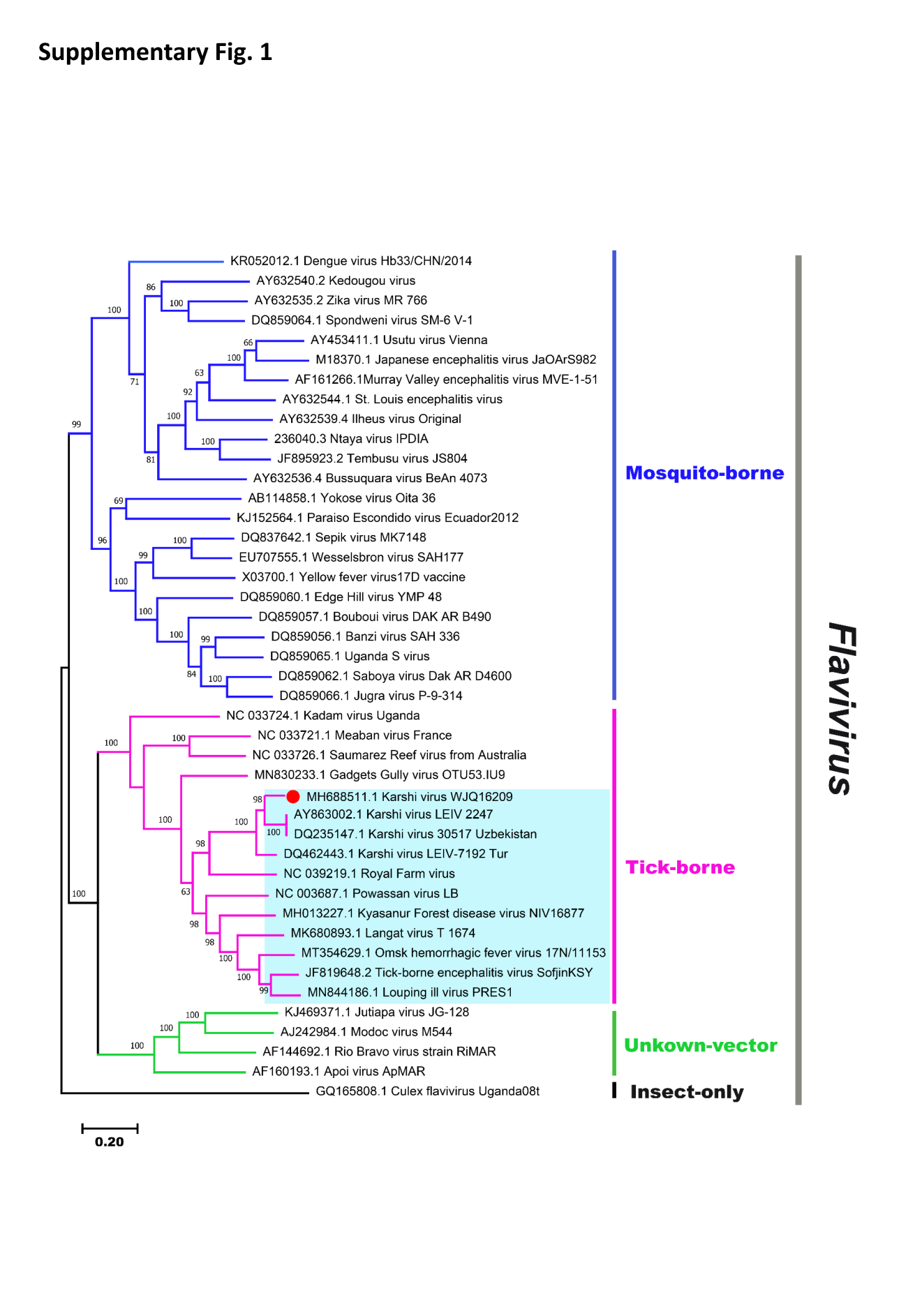
**

**Supplementary Figure 1.** Phylogenetic evolution analysis of flaviviruses built on the open reading frame of the polyprotein. The branches of four groups of flaviviruses with different hosts are marked with different colours. Karshi virus (KSIV) is labelled with a red solid circle, the turquoise shaded panel marks the tick-borne encephalitis virus (TBEV)-serocomplex group. The trees were constructed using Mega X and tested by the bootstrap method with 1000 replications. Bootstrap values greater than 60% are shown at each node.

**
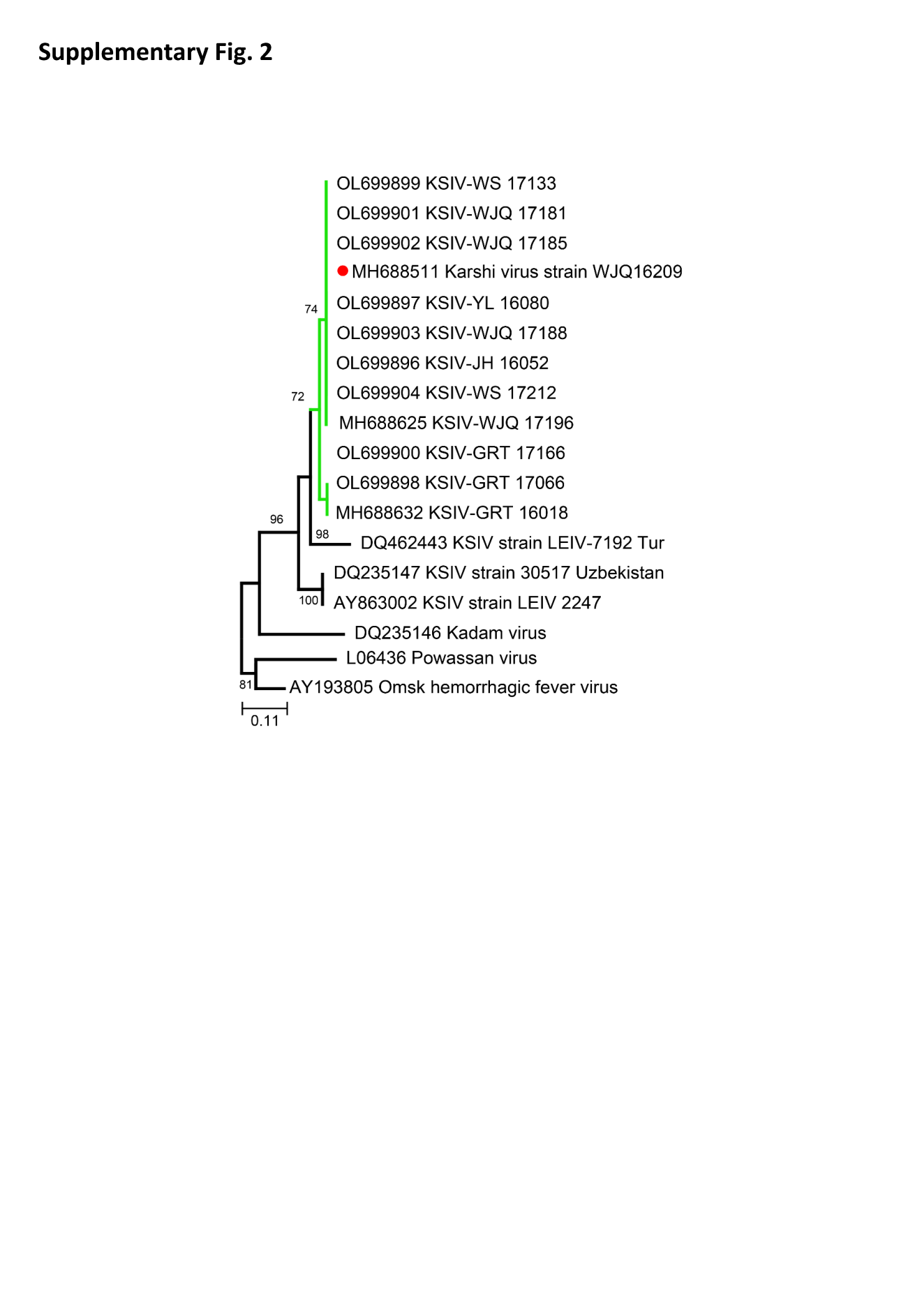
**

**Supplementary Figure 2.** Phylogenetic analysis of Karshi virus (KSIV) fragments in tick samples detected by PCR. Phylogenetic tree, showing relatedness based on 302 nucleotides of NS5 protein sequence, the main information in the evolutionary tree includes GenBank accession number, strain name. The green lines indicate the sequences obtained by RT-PCR, including tick group number, collection location and year with sample number, the red solid circle indicates Karshi virus strain WJQ16209. The trees were constructed using Mega X and tested by the bootstrap method with 1000 replications. Bootstrap values greater than 60% are shown at each node.

**
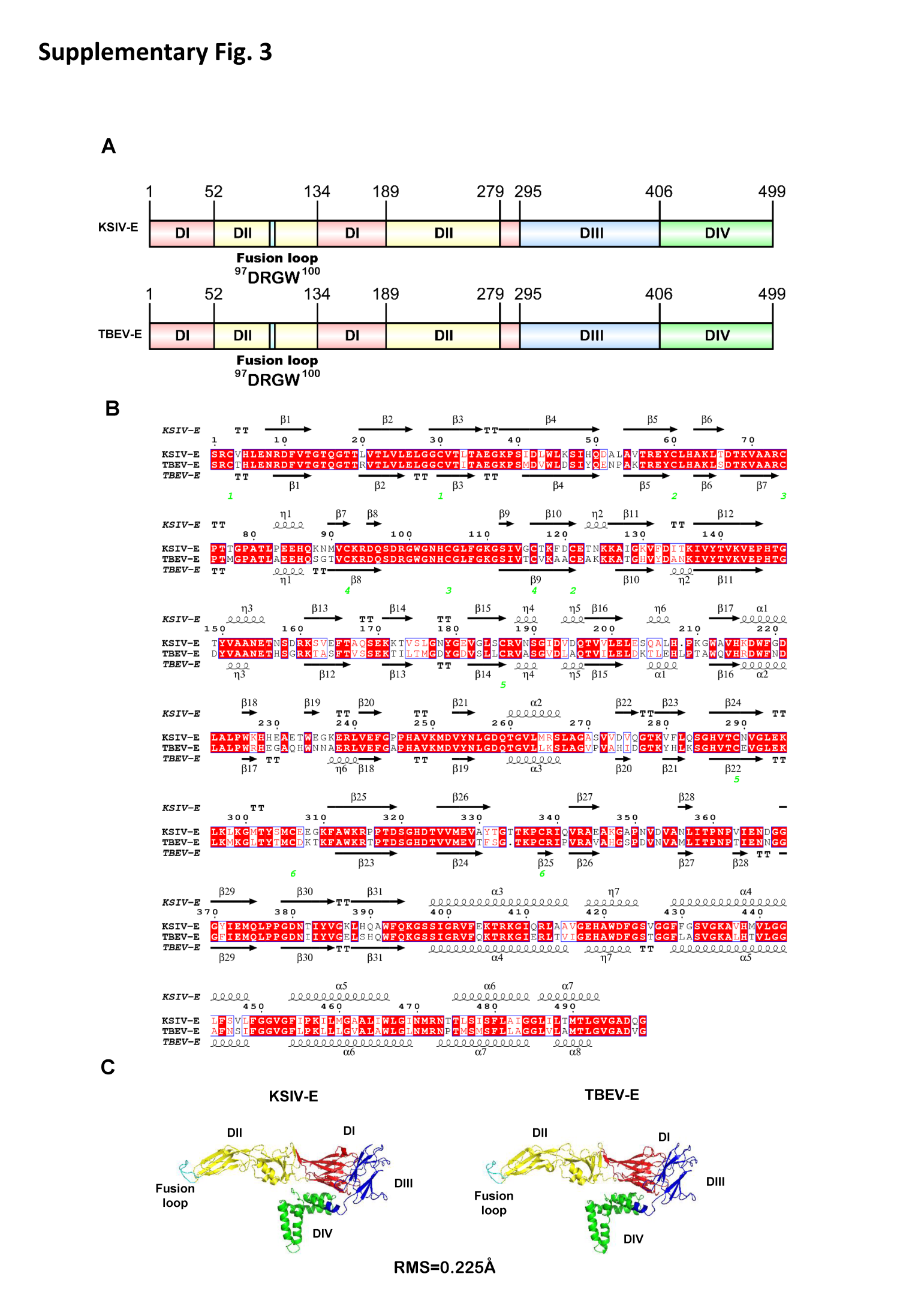
**

**Supplementary Figure 3.** Structures of Karshi virus (KSIV) and tick-borne encephalitis virus (TBEV) E proteins. (**A**) Schematic diagram of KSIV- and TBEV-E proteins. The domains are shown in different colours: red is DI, DII is in yellow, DIII is in blue, DIV is in green, the stem region in light blue is the highly conserved flavivirus fusion loop peptide “DRGW”. The numbers above the diagram represent the starting residue of each segment. (**B**) Sequence alignment of KSIV- and TBEV-E proteins. The axis represents the β-strand, the helices indicate the α-helix, TT is the turning residues of β-strands, and green numbers point out the disulfide bond-forming cysteines. The alignment map of KSIV- and TBEV-E proteins was generated using the ESPript (version 3.0) program and manually modified. (**C**) Comparison of the three-dimensional structure of the KSIV- and TBEV-E protein, domains are coloured as described in the legend to panel A. The predicted three-dimensional structure of KSIV- and TBEV-E protein was constructed by I-TASSER and visualized by Pymol^TM^ (version 1.4.1). The root-mean-square deviation (RMSD) was 0.225 Å.


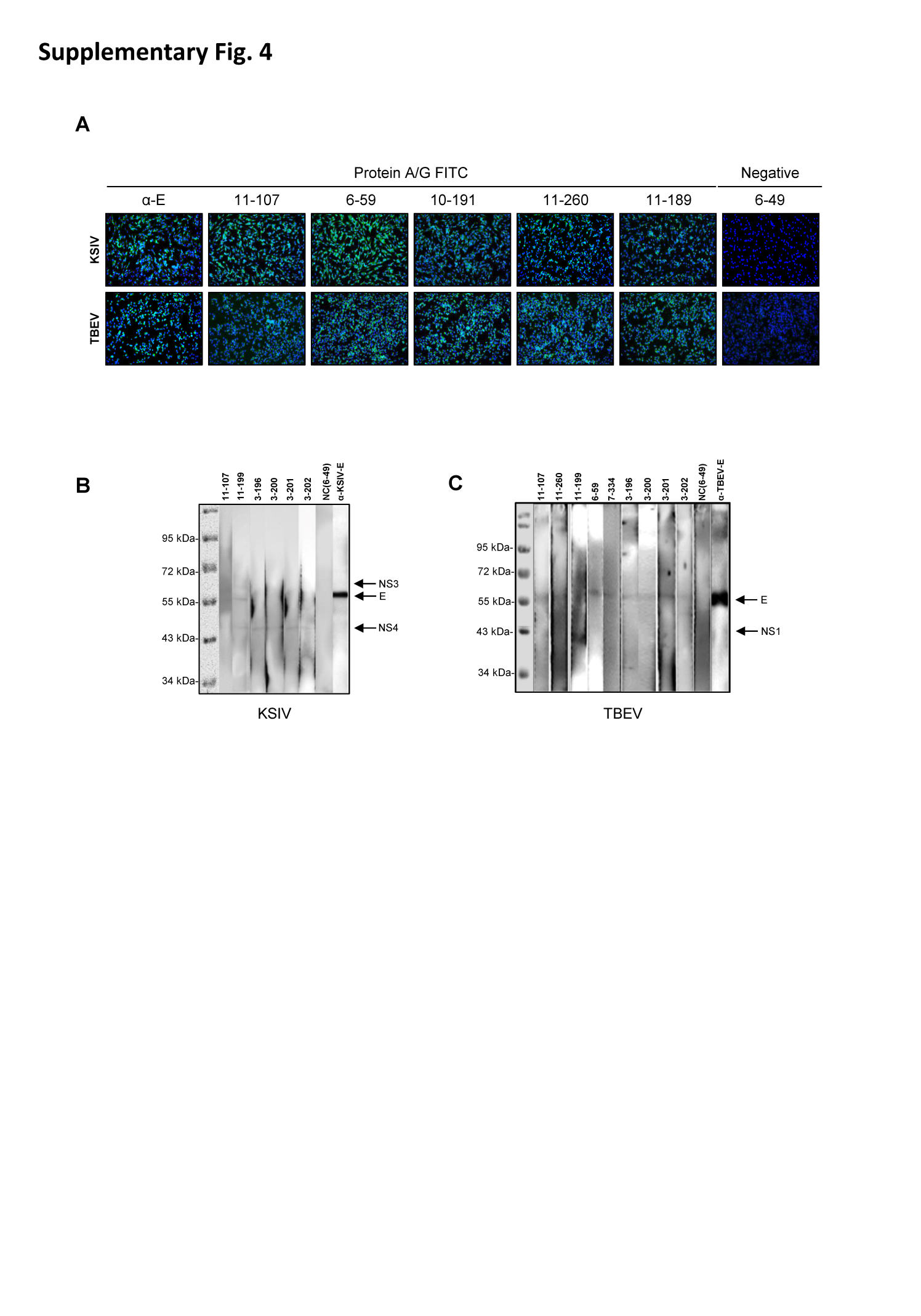


**Supplementary Figure 4.** Immunofluorescence and western blot results of Karshi virus (KSIV) and tick-borne encephalitis virus (TBEV) seroepidemiological tests. (**A**) Partial results of immunofluorescence detection of Xinjiang animal sera exposure to KSIV and TBEV. The BHK-21 cells were infected with KSIV or TBEV, then IFA was performed with the 11-107, 6-59, 10-191, 11-260 and 6-49 sera samples as the primary antibodies, and FITC-labelled Protein A/G as the secondary antibody; (**B**) and (**C**). Partial results of western blot analysis of IFA-positive animal serum. Purified KSIV and TBEV viruses were linearised as antigens, 11-107, 11-199, 3-196, 3-200, 3-201 and 3-202 sera samples were used as the primary antibodies for KSIV detection, while 11-107, 11-260,11-199, 6-59, 7-334, 3-196, 3-200, 3-201 and 3-202 sera samples were used as primary antibodies to detected TBEV, and α-E (α-KSIV-E or α-TBEV-E) were used as a positive control, sheep serum 6-49 was used as a negative control.

**Supplementary tables**

| Strain Name | GenBank  No. | KSIV WJQ16209-ORF  Identity (%) | | KSIV WJQ16209-E Identity (%) | |
| --- | --- | --- | --- | --- | --- |
|  |  | Amino acid | Nucleotide | Amino acid | Nucleotide |
| KSIV-LEIV-7192 | DQ462443.1 | 94.5 | 85.7 | 94.4 | 82.7 |
| KSIV-LEIV 2247 | AY863002.1 | 95.1 | 86.9 | 94.6 | 83.9 |
| KSIV-30517 | DQ235147.1 | 94.9 | 86.9 | 94.4 | 83.8 |
| POWV | NC_003687.1 | 70.5 | 67.2 | 72.2 | 56.9 |
| KFDV | MH013227.1 | 69.7 | 67.1 | 78.1 | 58.7 |
| LGTV | MK680893.1 | 70.0 | 67.3 | 70.8 | 56.0 |
| OHFV | MT354629.1 | 70.7 | 67.2 | 72.4 | 58.6 |
| LIV | MN844186.1 | 70.0 | 67.3 | 71.4 | 58.7 |
| TBEV | JF819648.2 | 71.1 | 67.9 | 78.3 | 58.1 |
| RFV | NC_039219.1 | 70.7 | 67.8 | 65.8 | 55.8 |

**Supplementary Table 1**. Amino acid and nucleic acid sequence identities of Karshi virus (KSIV) ORFs and E protein sequences compared with other Karshi virus (KSIV) strains as well as other members of the TBEV serocomplex group

KSIV, Karshi virus; POWV, Powassan virus; KFDV, Kyasanur Forest disease virus; LGTV, Langat virus; OHFV, Omsk haemorrhagic fever virus; LIV, louping ill virus; TBEV, tick-borne encephalitis virus; RFV, Royal Farm virus.

**Supplementary Table 2.** Detailed results of detecting antibody response and neutralization to tick-borne encephalitis virus (TBEV) using the Karshi virus (KSIV)-IFA positive animal serum samples

| Species | Location | No. |  | IFA | |  | WB | |  | LIPS* | |  | VNT | |
| --- | --- | --- | --- | --- | --- | --- | --- | --- | --- | --- | --- | --- | --- | --- |
|  |  |  |  | KSIV | TBEV |  | KSIV | TBEV |  | KSIV | TBEV |  | KSIV | TBEV |
| Marmot | Wuqia County | 3-247 |  | **+** | **+** |  | **-** | **-** |  | **-** | 1.90 |  | **-** | - |
|  |  | 3-195 |  | **+** | **+** |  | **+** | **+** |  | **-** | **-** |  | **-** | - |
|  |  | 3-196 |  | **+** | **+** |  | **+** | **+** |  | **-** | **-** |  | **-** | - |
|  |  | 3-199 |  | **+** | **+** |  | **+** | **+** |  | **-** | **-** |  | **-** | - |
|  |  | 3-200 |  | **+** | **+** |  | **+** | **+** |  | **-** | **-** |  | **-** | - |
|  |  | 3-201 |  | **+** | **+** |  | **+** | **+** |  | **-** | **-** |  | **-** | - |
|  |  | 3-202 |  | **+** | **+** |  | **+** | **+** |  | **-** | **-** |  | **-** | - |
|  |  | 3-203 |  | **+** | **+** |  | **+** | **+** |  | **-** | **-** |  | **-** | - |
|  |  | 3-204 |  | **+** | **+** |  | **+** | **+** |  | **-** | **-** |  | **-** | - |
|  |  | 3-205 |  | **+** | **+** |  | **+** | **+** |  | **-** | **-** |  | **-** | - |
|  |  | 1-2 |  | **+** | **-** |  | **-** | **-** |  | **-** | **-** |  | **-** | - |
|  |  | 3-281 |  | **+** | **-** |  | **-** | **-** |  | **-** | **-** |  | **-** | - |
|  |  | 4-c19 |  | **+** | **-** |  | **-** | **-** |  | **-** | **-** |  | **-** | - |
|  | Aheqi County | 11-107 |  | + | + |  | + | + |  | 1.00 | 2.76 |  | 2^5^ | 2^8^ |
|  |  | **11-260** |  | **+** | **+** |  | **-** | **+** |  | **1.37** | **-** |  | **2^6^** | - |
|  |  | **11-199** |  | **+** | **+** |  | **+** | **+** |  | **-** | **-** |  | **2^6^** | **2^4^** |
|  |  | **11-189** |  | + | + |  | - | - |  | **1.00** | **1.49** |  | **2^4^** | **2^4^** |
|  |  | 11-144 |  | **+** | **+** |  | **-** | **-** |  | **-** | 1.79 |  | **-** | - |
|  |  | **10-191** |  | **+** | **+** |  | **-** | **-** |  | **1.37** | **-** |  | **2^5^** | - |
|  |  | **10-258** |  | **+** | **+** |  | **-** | **-** |  | **1.34** | **-** |  | **2^5^** | **2^5^** |
|  |  | 11-101 |  | + | + |  | - | - |  | 1.31 | - |  | **-** | - |
|  |  | 11-145 |  | + | + |  | - | - |  | 1.00 | - |  | **-** | - |
|  |  | 11-37 |  | + | - |  | - | - |  | 1.05 | - |  | **-** | - |
|  |  | **10-264** |  | **+** | **-** |  | **-** | **-** |  | **1.24** | **-** |  | **2^5^** | - |
|  |  | 11-38 |  | **+** | **-** |  | **-** | **-** |  | **-** | **-** |  | **-** | - |
|  |  | 11-100 |  | **+** | **-** |  | **-** | **-** |  | **-** | **-** |  | **-** | - |
|  |  | 11-224 |  | **+** | **-** |  | **-** | **-** |  | **-** | **-** |  | **-** | - |
|  |  | 11-225 |  | **+** | **-** |  | **-** | **-** |  | **-** | **-** |  | **-** | - |
| Sheep | Qitai County | 6-59 |  | **+** | **+** |  | **-** | **+** |  | **-** | 2.70 |  | **-** | 2^6^ |
|  |  | 7-334 |  | **+** | **+** |  | **-** | **+** |  | **-** | 1.90 |  | **-** | 2^3^ |
|  |  | 7-168 |  | **+** | **+** |  | **-** | **-** |  | **-** | 1.16 |  | **-** | 2^7^ |
|  |  | 7-177 |  | **+** | **+** |  | **-** | **-** |  | 1.37 | **-** |  | **-** | 2^5^ |
|  |  | **6-132** |  | **+** | **+** |  | **-** | **-** |  | **-** | **-** |  | **2^4^** | - |
|  |  | 7-289 |  | **+** | **+** |  | **-** | **-** |  | **-** | **-** |  | **-** | - |
|  |  | 8-289 |  | **+** | **-** |  | **-** | **-** |  | **-** | **-** |  | **-** | - |
|  |  | 7-293 |  | **+** | **-** |  | **-** | **-** |  | **-** | **-** |  | **-** | - |

IFA, immunofluorescence assays; WB, western blot assays; fold change to the *LIPS cut-off value; VNT, virus neutralization titer; +, positive for antibodies; –, negative for antibodies.

The samples that were likely to have TBEV infection and show cross-reaction to KSIV are shaded; the samples that were likely to have KSIV infection and show cross-reaction to TBEV are shown in bold; the samples that were likely to have co-exposure to both viruses or might have been infected with one of these two viruses and present cross-reaction to the other are shown in bold, shaded, and boxed.
